# Ridge mini-implants, a versatile biomechanical anchorage device whose success is significantly enhanced by splinting: a clinical report

**DOI:** 10.1186/s40510-023-00480-5

**Published:** 2023-08-28

**Authors:** Sarah Abu Arqub, Renee Greene, Sara Greene, Kolbe Laing, Chia-Ling Kuo, Lucas Da Cunha Godoy, Flavio Uribe

**Affiliations:** 1 Department of Orthodontics, University of Florida, Gainesville, FL USA; 2 Division of Orthodontics, Department of Craniofacial Sciences, University of Connecticut Health, Farmington, CT USA; 3 UCONN School of Dental Medicine, University of Connecticut, Farmington, CT USA; 4 Connecticut Convergence Institute for Translation in Regenerative Engineering, UConn Health, Farmington, CT USA

**Keywords:** Alveolar ridge mini-implants, Clinical success, Length, Splinting

## Abstract

**Objectives:**

This clinical report aims to highlight the factors affecting the clinical success of alveolar ridge mini-implants used for orthodontic anchorage and provide an overview of the biomechanical versatility of this miniscrew and steps involving the proper technique of its placement.

**Methods:**

For this clinical report, charts for 295 patients who had temporary anchorage devices (TADs) were screened. Twenty patients [15 females and 5 males: mean age = 38.15 ± 15.10 years] with 50 alveolar ridge mini-screws were assessed. A descriptive summary of the main factors affecting their clinical success and the technique employed for their placement was comprehensively discussed and illustrated, in addition to the presentation of some clinical cases illustrating their potential clinical uses.

**Results:**

The survival duration (7.32 ± 9.01 months) and clinical success of the alveolar ridge mini-implants that failed (19/50) seem to be affected primarily by 2 factors: splinting; none of the splinted mini-implants failed (0/10) compared to (19/40) of the single mini-implants that failed, and the length of the used mini-implant; the average length of the mini-implants that did not fail was 9.23 mm. Additionally, it appears that these mini-implants are biomechanically robust and durable, those that did not fail had an average survival duration of 35.97 ± 19.79 months.

**Conclusion:**

Ridge mini-implants offer significant biomechanical versatility in patients with partially edentulous ridges needing complex pre-prosthetic orthodontic movements. The presence of splinting and the length of the used mini-implants are factors that might affect the clinical success of the alveolar ridge mini-implants.

## Introduction

Mini-implants have been extensively described as temporary anchorage devices TADs with multiple applications in orthodontics and orthopedics [1–5]. The applied biomechanics relies on bone as the anchor unit to drive orthodontic tooth movement [6]. Also termed mini-screws, these TADs have been successfully placed in various inter-radicular and extra-alveolar sites. The highest survival rate was reported for the palatal mini-implants (91.5%), while the lowest was reported for those placed in the buccal shelf area (31.3%) [7]. Therefore, palatal mini-implants can be considered the gold standard with regard to long-term survival and stability [7].

However, when orthodontic tooth movement is planned for an orthodontic patient with a long span partially edentulous ridge, understanding of important biomechanical principles to enhance anchorage is often required [8, 9]. The large interdental edentulous segments hamper the biomechanical control of tooth movement needed to protract a tooth distal to an edentulous area. Moreover, if retraction of an anterior segment is planned, the use of mini-implants in the buccal surface of the alveolar ridge would constitute a complex biomechanical challenge on planning and controlling the direction of orthodontic forces required for retraction.

Previously, osseointegrated dental implants were suggested to provide skeletal anchorage solution to aid in orthodontic tooth movement in multidisciplinary cases for partially edentulous patients [10]. They served as assets in improving inter and intramaxillary absolute anchorage for partially edentulous orthodontic patients [11]. Limitations related to their placement in the initial stages of orthodontic treatment might compromise the precise space appropriation for the final planned restoration in the three planes of space [12]. That being the case, a recommended solution to aid in the biomechanical control of orthodontic tooth movement in these partially edentulous subjects, is the alveolar ridge mini-implant. These mini-implants are often temporarily placed on the alveolar ridge in the edentulous site. This allows the possibility for their removal once their desired function is achieved and for the appropriate placement of the final planned restoration in the three planes of space at the end of orthodontic treatment. These ridge mini-implants have been reported only in a couple of case reports [8, 9]. Their use was primarily indicated for pre-prosthetic molar uprighting. Their advantage lies in that they can act as a temporary tooth and provide better force control and a stable anchorage for molar uprighting [8]. Therefore, this clinical report aims to illustrate their biomechanical uses and give an overview of some factors that might influence their clinical success.

## Clinical benefits and uses of the alveolar ridge mini-implants

**Fig. 1:**
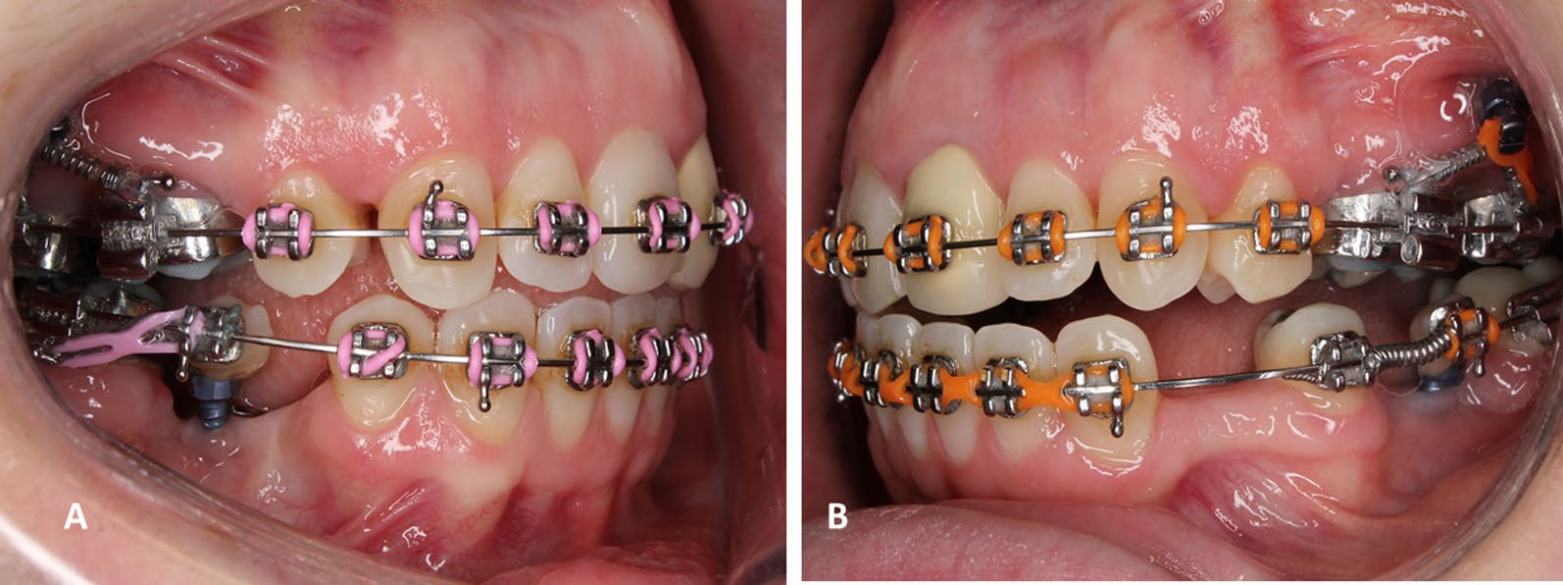
Push and pull-type force delivery from ridge mini-implants. **A** Protraction force (pull-type) to mesialize mandibular second molar delivered from a ridge mini-implant into a large edentulous space. **B** Premolar protraction force (push-type) for space appropriation in edentulous sites from a ridge mini-implant

**Fig. 2:**
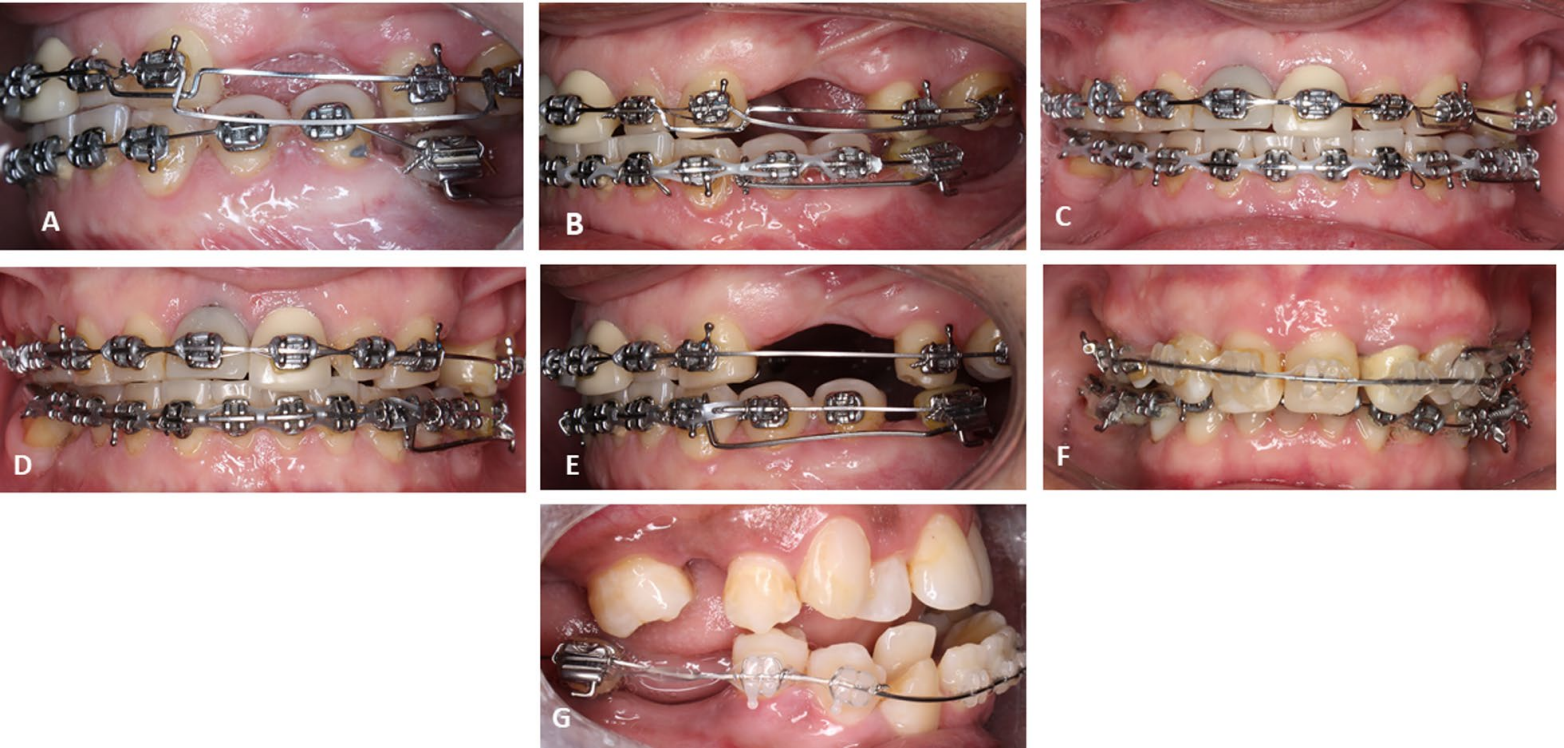
Versatility of different force delivery systems from ridge mini-implants. **A** Intrusion of an adjacent premolar to the ridge mini-implant that had supraerupted into the edentulous antagonistic maxillary premolar region. Intrusive force delivered by means of a deflected nickel-titanium wire. **B** Post-intrusion occlusal relationship. **C** Transverse force delivery with a cantilever system driven from an alveolar ridge mini-implant in an edentulous left first molar site. **D** Midline correction by means of this transverse force. **E** Canine transverse correction with lingually directed force delivery from the cantilever system observed in **C** and **D**. **F** Asymmetric force delivery from a ridge mini-implant in the left premolar region for incisor plane cant correction by means of an intrusive force from a cantilever system. **G** Retraction of the anterior teeth from a posterior fully edentulous site with a ridge mini-implant

**Fig. 3:**
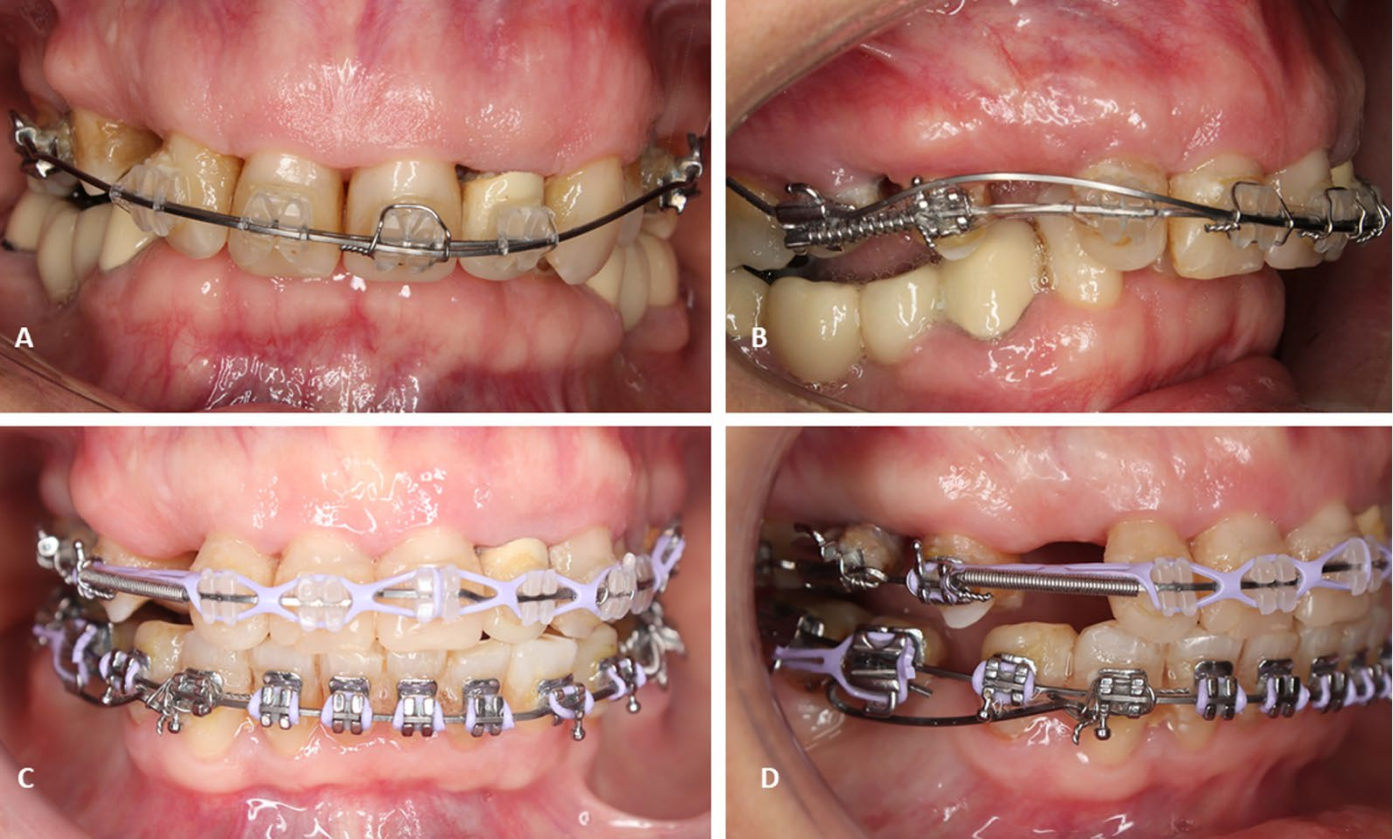
Intrusion arch delivered from ridge mini-implants in premolar/molar region. **A** Pre-intrusion overbite frontal view (**A**) and lateral view (**B**). **C** Post-intrusion overbite correction, frontal view and lateral view (**D**)

**Fig. 4:**
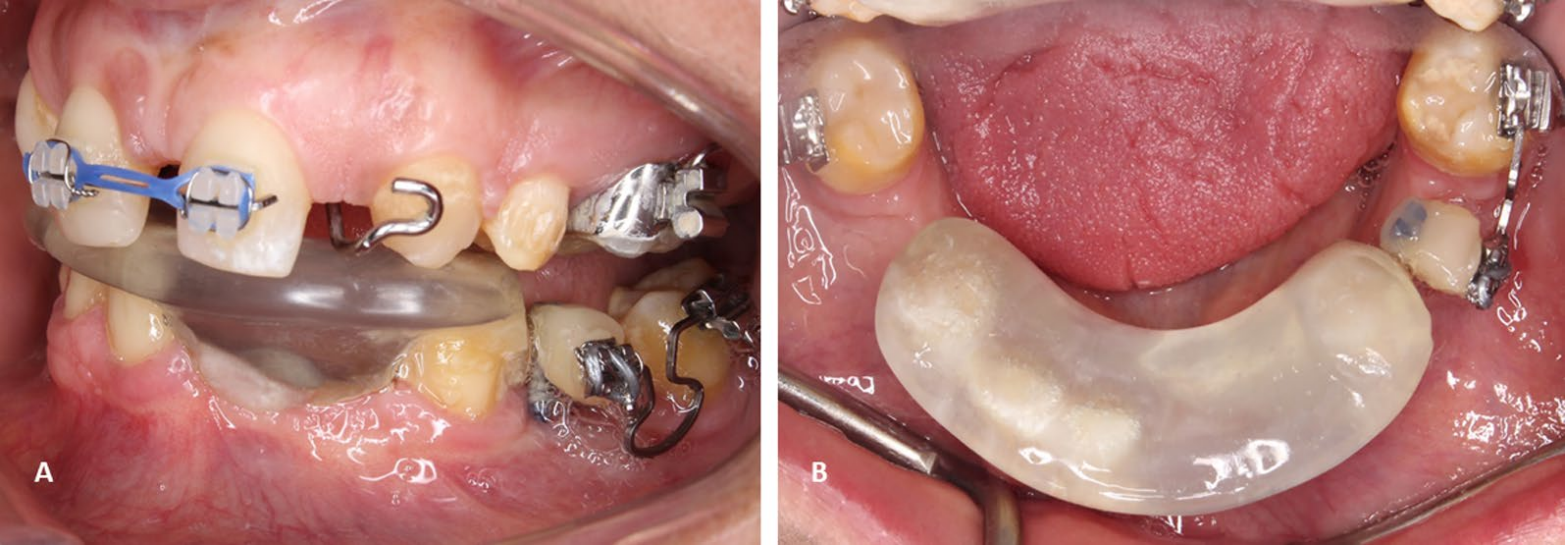
Frictionless segmental mechanics for molar protraction from ridge mini-implant with a T-loop. **A** Lateral view, **B** frontal view

From a biomechanical perspective, rigid sliding tracks with the use of palatal mini-implants were suggested to protract maxillary teeth along an edentulous span [13]. Results have shown that they provide adequate anchorage for controlled tooth movement in the anteroposterior plane [14]. However, in the vertical plane, the biomechanical possibilities for controlled vertical tooth movement while drawing anchorage from palatal mini-implants are often restricted. Further, the use of the these sliding tracks for protraction is not feasible in the mandibular arch due to anatomical constraints. Therefore, ridge mini-implants offer a solution for a controlled tooth movement over these large edentulous sites in various planes. In addition, they can profitably provide a robust posterior anchor to retract anterior teeth in partially edentulous patients, as an alternative to the use of the osseointegrated dental implants. Moreover, another added value that is exclusive to the use of alveolar ridge mini-implants, is that once inserted into the alveolar ridge, flowable composite resin can be added on top of these mini-implants and shaped as a tooth-like structure, to which a bracket and/or tube can be attached. For that reason, these mini-implants can be incorporated into the main archwire. This offers the distinctive ability to deliver push- and pull-types of forces simultaneously (Fig. 1). This is considered crucial since majority of mini-implant force delivery systems rely on a pull mechanism to move teeth. Consequently, due to their above-mentioned advantages, various biomechanical movements can be driven with their use such as: intrusion of adjacent and distant teeth (Fig. 2A, B), lateral movement (transverse) (Fig. 2C–E), incisal/occlusal cant correction (Fig. 2F), protraction (Fig. 1), retraction (Fig. 2G), and distalization. On the other hand, in the vertical plane, different designs and lengths of extended cantilever arms can be attached to these alveolar ridge mini-implants to allow vertical movement of adjacent and distant teeth (Fig. 3). Finally, segmental frictionless mechanics such as loops can be delivered from these TADs in the alveolar ridge (Fig. 4).

## Descriptive summary of the clinical cases with the alveolar ridge mini-implants

Twenty subjects [15 females and 5 males: mean age = 38.15 ± 15.10 years] had ridge mini-implants placed between January 2010 and February 2022 at University of Connecticut Health - Division of Orthodontics. Six of them had one mini-implant, seven had two mini-implants, and the last seven had more than 2 and up to 7 mini-implants placed in their alveolar ridges. Overall, 50 alveolar ridge mini-implants were placed and assessed for this descriptive clinical report. A mini-implant was considered failed once it no longer fulfilled its purpose or when it was completely absent. The time in months from placing the mini-implant to failure was tracked for those that failed (7.32 ± 9.01 months); further, end of use of the mini-implant or follow up (February 2022) for those that did not fail (35.97 ± 19.79 months). The diameter of the inserted mini-implants varied between 1.5- 2.3 mm, with a length that ranged between 7 and 17 mm. Majority of these mini-implants were inserted by a faculty member and some by residents. The conventional manual self-drilling technique with a contra-angle was used for their insertion as described below:

1. Panoramic radiographs are used to estimate the location of the inferior alveolar nerve in the mandible and the location of the maxillary sinus prior to mini-implant placement.
2. Small volume cone beam computed tomography (CBCT) images (Planmeca ProMax 3D, (scan time: 9–40 s; field of view [FOV]: 5 × 5.5 cm up to 23 × 26 cm and voxel size: 75–600 µm) are captured in cases where the alveolar ridge height is reduced, to avoid nerve injury in the mandible.
3. Clinical evaluation for the width of the alveolar ridge, in cases of a knife-edge ridge, a pilot hole is recommended for use.
4. 3M™ Peridex™ Chlorhexidine Gluconate 0.12% oral rinse, followed by topical and local anesthesia, is administered prior to placement.
5. Contra-angle screw drivers are used for the insertion of the mini-implants.
6. Considerations during insertion:
  Ridge anatomy in the coronal plane (the mini-implant should be parallel to an imaginary vertical line that passes through the central fossa of adjacent teeth).
  The mini-implant attachment head should be positioned vertically out of occlusion, and transversely referencing the buccal surface of the adjacent teeth to avoid excessive wire bending.
7. Flowable composite is used to build the attachment head to replicate a crown of a tooth
8. If the purpose for the alveolar ridge mini-implant is for push and pull mechanics along the arch or a cantilever mechanics are planned, a double tube is attached to the buccal surface of the built composite.
9. Wires are bent passively to engage the mini-implants, occlusion evaluated, and the mini-implants is used for the above-mentioned purposes.

**Fig. 5:**
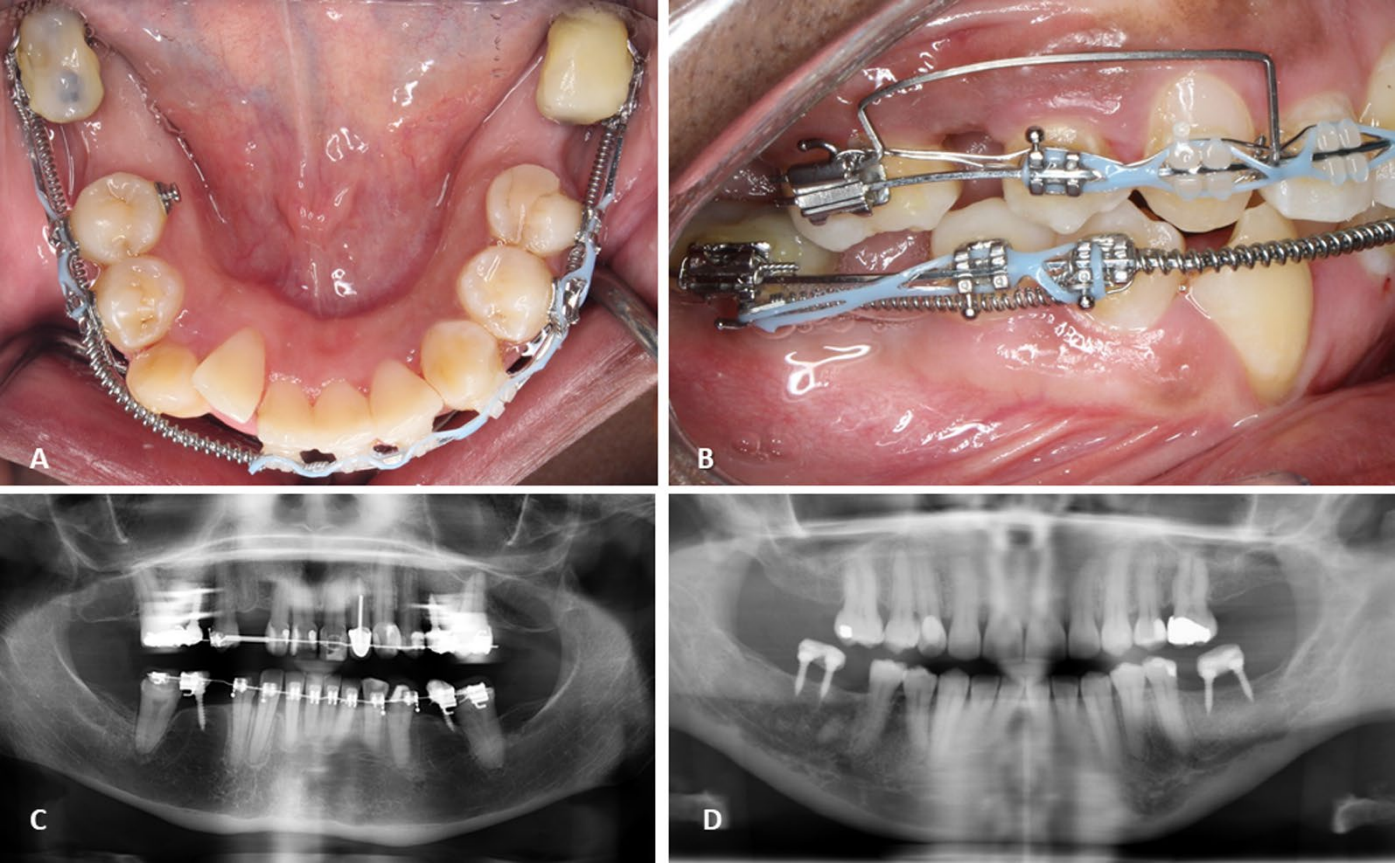
Ridge mini-implants clinical and radiographic examples. **A** Splinted ridge mini-implants in the mandibular edentulous region. **B** Lateral view of the splinted mini-implants used for retraction of the premolar region. **C** Panoramic radiograph depicting single mini-implants, one on each quadrant. **D** Panoramic radiograph of clinical photograph in A and B showing splinted mini-implants

Figure 5 shows an example of ridge mini-implants which involves a self-drilling approach. The mini-implant head is typically built up in the shape of a temporary tooth with flowable composite, and a bracket is bonded to the facial surface in order to incorporate the orthodontic main archwire. Factors that affected the time to failure seemed to be mainly related to the presence of splinting and the length of the inserted mini-implants. All the mini-implants that were splinted had no failure (10 units, 100% success) compared to 52.5% (21 units) success in single mini-implants. The average length of the mini-implants that did not fail was 9.23 mm, while those that failed had a length of 8.55 mm.

## Discussion

With the increased use of mini-implants for various applications in orthodontics [15, 16], different sites have been recommended for their safe insertion, including the intra-alveolar vestibular locations between the roots [17], palatal [18] and extra-alveolar sites such as the buccal shelf and the infrazygomatic regions [19]. Despite the popularity of the above-mentioned locations for mini-implants’ insertion, the intra-alveolar vestibular mini-implants being the most popular and convenient suffer from limitations related to the restricted interradicular spaces due to proximity between the adjacent roots, which might increase the risk of damaging the roots and periodontium, and lead to an early loss of the mini-implant (high possibility of failure) due to root contact and the high potential for its fracture if pushed against the root [20]. This clinical report is innovative in further suggesting and exploring a new site (the alveolar ridge) for inserting orthodontic mini-implants for various applications as an alternative to the previously mentioned locations. The main indication for this location is for patients with edentulous areas that will be optimized preprosthetically.

The alveolar ridge mini-implants provide a good alternative to other mini-implant locations for multidisciplinary treatment. For that reason, patients in this clinical report were adult patients (average age: 38.15 ± 15.10 years). These mini-implants can act as temporary teeth, which are often built using composite filling materials, bracketed and behave as anchor units (similar to a dental implant) for various types of desired orthodontic tooth movement. Unlike dental implants, they do not require extensive surgery for their placement, nor a lengthy healing time to allow for osseointegration. Additionally, they do not impose financial concerns to the patients and due to their small size, they can be easily and safely placed and replaced as needed in various anatomical locations [10].

Splinting and length of the used mini-implants seems to affect the alveolar ridge mini-implants’ survival. Chen et al. found a significant relationship between success rate and the length of mini-implants [21]. Tseng et al. showed that the success rate is often increased with greater mini-implant length, yet results were not statistically significant [22]. Similarly, the length of the alveolar ridge mini-implants seems to influence their survival rate and its durability; therefore, it is recommended to insert longer mini-implants in the alveolar ridge area. On the other hand, the operator’s experience in placing the mini-implant might also have an effect on its failure [23]. Finally, the success rate dramatically increased when two mini-implants were splinted together and loaded as one unit. There might be a possibility that splinting reduces the amount of stresses by distributing the load over a larger area. This should be taken into consideration when planning force vectors and biomechanics. Patients are often treated with a single mini-implants for anchorage; splinting was often done to ensure greater stability. It is a technique that often involves placing 2 alveolar ridge mini-implants adjacent to each other and adding flowable composite on both of their head attachments. Figure 5A, B, and D depicts the splinting of 2 mini-implants. Similarly, this technique has been advocated previously with the use of palatal mini implants, where splinting was done by bonding the S-sheath on the top of 2 mini-implants with flowable composite resin, and showed higher success rate (95.9%) than using a single mini-implant (82.4%) in the palate [23, 24]. This suggests that stability can be enhanced with the increased length of the alveolar ridge mini-implant and splinting. Therefore, it is recommended to splint these ridge mini-implants and use a mini-implants of at least a 10 mm in length. In some instances, splinting may be difficult due to ridge dimensions. In those situations, single mini-implants with 10 mm in length may provide acceptable stability although some degree of mini-implant failure would be expected. Despite the failure of some single units, the versatility and biomechanical advantages of this mini-implant make it an ideal resource for patients with long edentulous spans requiring pre-prosthetic orthodontic treatment.

## Conclusion and recommendations

This clinical report shows that alveolar ridge mini-implants are considered a versatile and powerful biomechanical temporary anchorage tools. Patients with long edentulous spans requiring pre-prosthetic orthodontic treatment can greatly benefit from these mini-implants. Overall, the clinical success of the alveolar ridge mini-implants might be affected by the length and the use of 2 splinted alveolar ridge mini-implants with composite resin.

## Data Availability

The data underlying this article are available in the article and in its online material.

## Abbreviations

TAD: Temporary anchorage devices
CBCT: Cone beam computed tomography
